# Insulin receptor alternative splicing is regulated by insulin signaling and modulates beta cell survival

**DOI:** 10.1038/srep31222

**Published:** 2016-08-16

**Authors:** Pushkar Malakar, Lital Chartarifsky, Ayat Hija, Gil Leibowitz, Benjamin Glaser, Yuval Dor, Rotem Karni

**Affiliations:** 1 Department of Biochemistry and Molecular Biology, The Institute for Medical Research Israel-Canada, Hebrew University-Hadassah Medical School, Ein Karem, 91120, Jerusalem, Israel; 2 Department of Developmental Biology and Cancer Research, The Institute for Medical Research Israel-Canada, Hebrew University-Hadassah Medical School, Ein Karem, 91120, Jerusalem, Israel; 3 Endocrinology and Metabolism Service, Department of Medicine, Hebrew University-Hadassah Medical School, Ein Karem, 91120, Jerusalem, Israel

## Abstract

Type 2 Diabetes (T2DM) affects more than 300 million people worldwide. One of the hallmarks of T2DM is peripheral insulin resistance, in part due to unproductive insulin signaling through the insulin receptor. The insulin receptor (*INSR*) exists as two isoforms, INSR-A and INSR-B, which results from skipping or inclusion of exon 11 respectively. What determines the relative abundance of the different insulin receptor splice variants is unknown. Moreover, it is not yet clear what the physiological roles of each of the isoforms are in normal and diseased beta cells. In this study, we show that insulin induces *INSR* exon 11 inclusion in pancreatic beta cells in both human and mouse. This occurs through activation of the Ras-MAPK/ERK signaling pathway and up-regulation of the splicing factor SRSF1. Induction of exon 11 skipping by a splice-site competitive antisense oligonucleotide inhibited the MAPK-ERK signaling pathway downstream of the insulin receptor, sensitizing the pancreatic β-cell line MIN6 to stress-induced apoptosis and lipotoxicity. These results assign to insulin a regulatory role in *INSR* alternative splicing through the Ras-MAPK/ERK signaling pathway. We suggest that in beta cells, INSR-B has a protective role, while INSR-A expression sensitizes beta cells to programmed cell death.

The endocrine pancreas is a dynamic organ capable of responding to challenges by balancing β-cell proliferation, differentiation and apoptosis^1^. Low levels of ‘physiological’ apoptosis are a normal part of islet maintenance. However, under certain circumstances the rate of β-cell death may outpace the rate of β-cell birth. In T2DM and animal models of diabetes, β-cell apoptosis is increased^2^,^3^. In many tissues, cell fate and function are regulated by autocrine/paracrine signals. There is now strong evidence that insulin is an essential regulator of β-cell mass. Mice lacking β-cell insulin receptors (βIRKO) have increased apoptosis, decreased proliferation and reduced β-cell mass^4^. Furthermore, the compensatory β-cell proliferation normally seen in response to high fat diet induced obesity and hyperinsulinemia was completely absent in βIRKO mice. The observation that transgenic re-expression of insulin receptors in β-cells partially rescued mice with global insulin receptor knockout further supports the idea that β-cells are one of the most important insulin target tissues^5^. It has been recently shown that insulin directly prevents apoptosis in human and mouse islets^6^, as well as in β-cell lines and it also directly stimulates replication in primary β-cells^7^. The insulin signaling network contains many proteins that are established regulators of apoptosis and proliferation. For example, insulin receptor substrate 2 (*Irs2*) knockout mice displayed increased β-cell apoptosis^8^,^9^. This suggests that insulin signaling, through specific pathways, can exert selective effects on β-cell survival. The Akt arm of the insulin signaling pathway has been well studied. Islets lacking 80% Akt activity have been found to have normal β-cell mass and still respond to the protective effects of insulin. Raf-1, which belongs to the Ras-MAPK pathway arm of the insulin signaling pathway, has a critical role in β-cell survival and proliferation^7^,^10^. Undoubtedly, understanding key events in these pathways is crucial for any effort to harness the protective effects of autocrine insulin signaling for the treatment of diabetes.

Alternative splicing is a key process that produces different proteins from the same gene. Recently, alternative splicing has been shown to play a role in various diseases such as cancer and to regulate the Ras-MAPK and the PI3K-mTOR signaling pathways^11^.

The insulin receptor protein has two isoforms generated by alternative splicing of the *INSR* gene^12^,^13^. Isoform B (INSR-B), which includes exon 11, is associated with stronger insulin binding, while isoform A (INSR-A), which excludes this exon, binds both insulin and IGF-II^14^. The different *INSR* isoforms differ also in their ability to activate downstream signaling pathways^15^. The importance of the different isoforms for pancreatic β-cell proliferation or survival is unknown and the factors that determine which isoform will be formed in the cell are also largely unknown^16^. Alteration of *INSR* alternative splicing has been associated with several diseases such as myotonic dystrophy^12^,^17^, cancer^18^, and recovery from type 2 diabetes after bariatric surgery^19^.

Insulin receptor alternative splicing is cell specific, and the relative proportions of the different isoforms vary during development, aging and different disease states^20^. In adult life, INSR-A is ubiquitously expressed, whereas INSR-B is predominantly expressed in pancreatic beta cells, liver and also in muscle, adipose tissue, and kidney, which are all target tissues of the metabolic effects of insulin^21^. The predominant isoform in human pancreatic β-cells is INSR-B^22^. INSR-A is predominantly expressed in prenatal life and has a much higher affinity for IGF-II, and thus INSR-A plays an important role in fetal development and may play a role in certain cancers, while INSR-B is more involved in ‘metabolic’ signaling^14^. Furthermore, INSR-A has a faster internalization and recycling time, and also an overall lower signaling capacity, including a twofold lower tyrosine kinase activity^23^. IGF-I also binds better to INSR-A, but not as well as IGF-II^24^. Despite the importance of INSR biology in beta cells, what determines the relative abundance of the different insulin receptor splice variants remains mostly unknown. A high INSR-A/INSR-B ratio has been implicated in the insulin resistance of patients with myotonic dystrophy and possibly in patients with T2DM^13^. The sequences involved in *INSR* exon 11 splicing are only partially known. Transient transfection experiments in human hepatocellular carcinoma cells (HepG2) with *INSR* minigenes spanning exon 10 to 12 allowed for identification of a 48-nucleotide purine-rich sequence at the 5′ end of intron 10 that functions as a splicing enhancer and increases exon 11 inclusion^25^. Moreover, a 43-nucleotide sequence that favors skipping of exon 11 has been mapped upstream of the break point sequence of intron 10. Recently, over-expression and knockdown studies with hepatoma and embryonic kidney cells demonstrated that SRSF3 and SRSF1 (previously known as SRp20 and SF2/ASF, respectively) bind to the exonic splicing enhancers in exon 11, increasing exon inclusion, but that CUG-binding protein CUG-BP1 (CELF1) causes exon skipping by binding an exonic silencer. Therefore, the relative ratios of SRSF3 and SRSF1 to CELF1 in different cells determine the degree of exon inclusion^26^. Moreover, linker scanning mutations in intron 10 identified AGGGA sequences that are important for enhancer function. Using RNA-affinity purification and mass spectrometry, hnRNP F and hnRNP A1 were found to bind these sites and also to similar motifs at the 3′ end of the intron. The hnRNPs have opposite functional effects on exon 11 inclusion, hnRNP F promoting and hnRNP A1 inhibiting inclusion^27^. The complexity of splicing factor interplay involved in the regulation of tissue-specific or developmental-related exon 11 skipping remains unclear^28^.

In this study we show that insulin, through activation of the Ras-MEK1-ERK pathway, elevates the levels of splicing factor SRSF1 and induces inclusion of INSR exon 11. We further show that elevated levels of splicing isoform INSR-B are protective for beta cells, as antisense oligos that induce exon 11 skipping sensitize beta cells to stress-induced apoptosis.

## Materials and Methods

### Cells

MIN6 (Mouse insulinoma cells) were cultured in DMEM with 15% FCS, 2 mM L-Glutamine, 0.1 mg/ml penicillin, 0.1 mg/ml streptomycin and 50 μM β-mercaptoethanol. Cadaveric human islets were cultured in RPMI-1640 with 10% HI-BS, 2 mM L-Glutamine, 0.1 mg/ml penicillin, 0.1 mg/ml streptomycin. Mouse islets were isolated as described^29^ and cultured in RPMI-1640 with 10% HI-BS, 2 mM L-Glutamine, 0.1 mg/ml penicillin, 0.1 mg/ml streptomycin. Human islets were obtained as described^29^.

### Handling human islets

Cadaveric human islets were obtained from University of Edmonton, Canada. In order to prepare them for use, the medium in which they arrived was removed (after letting the cells sink to the bottom for 20 minutes). Cells were resuspended in 3 ml RPMI-1640 supplemented with 10% heat inactivated adult bovine serum and passed through a 7 μM filter. The cells were transferred to a non-tissue culture petri dish (Langerhans islets are usually cultured in suspension where they do not attach) with the appropriate medium and incubated over night at 37 °C prior to use.

Cadaveric human islets were obtained under the approval of the University of Edmonton Ethics committee and in accordance to the guidelines of University of Edmonton, Canada. A written informed consent was obtained from all subjects.

### Isolation of mouse islets

Wild type mice were sacrificed and the pancreas was dissected according the following protocol. First, the common bile duct (CBD) was clamped off near the junction with the small intestine. The CBD was then cannulated with a 27–30 gauge needle at the junction of the cystic duct from the gall bladder and left hepatic duct from the liver. 5 ml of cold HBSS containing 1.5 mg/ml Collagenase-P and 10 μg/ml DNase I were injected into the CBD. The pancreas was then removed, placed into a 10 cm petri dish and incubated for 6 min at 37 °C. Next, the pancreas was mechanically dismantled by pipetting up and down with 10 ml of cold HBSS/DNase I and islets were hand-picked under a dissecting microscope into a new petri dish containing HBSS/DNase I. Collection was repeated until islets were clean from dissembled cells and cells from other tissues. After the last collection islets were transferred into an eppendorf tube containing HBSS without DNase I. Islets were centrifuged at 1000 rpm and HBSS was removed. Finally, islets were seeded into dishes for suspension cultures where they do not attach with the appropriate medium. Islets were incubated at 37 °C over night for recovery and experiments were conducted on the following day.

All animal experiments were approved by the Hebrew University ethics committee for animal experiments and performed in accordance with the guidelines of the Hebrew University committee for the use of animals for research.

### Antisense oligonucleotides (ASOs)

2′-OMe antisense oligonucleotides (ASO) with Cy5 at the 5′ end (Sigma) were used to induce skipping of *INSR* exon 11. INSR ASO sequence was designed against intron 10-exon 11 junction of the human *INSR*, with one mismatch in the mouse intron (T instead of C): 5′-GCCTGAAGAGGTTTTTCTGTGGAA. A scrambled (SCR) ASO which does not base-pair with any known genes was used as negative control. SCR ASO sequence: 5′-GCAAUCCGCAAUCCGCAAUCC. For transfection, 5 × 10^5^ MIN6 cells/well in 6 well plates were seeded and transfection was performed on the following day (cells were ~70% confluent). INSR and Scrambled ASO working concentrations were 2.5 μM in a final volume of 700 μl Opti-MEM medium. 7.0 μl of Lipofectamine2000 was used per 2.5 μM ASO. Transfection was according to the manufacturer’s instructions (work with ASO was done in a dark hood).

### Palmitate and Glucotoxicity assays

MIN6 cells were incubated in normal MIN6 medium with 0.5% (w/v) BSA with or without 0.5 mM palmitate for 48 hours. The palmitate-BSA solution was prepared as follows. Briefly, the sodium salt of palmitic acid was dissolved at a concentration of 10 mM in 11% BSA in a shaking water bath at 37 °C for 16 hours. The palmitate-BSA solution was adjusted to pH 7.4 with 1 N NaOH, filtered through a sterile 0.22 micron filter and stored at −20 °C. The palmitate-BSA solution was diluted 1:20 in the MIN6 incubation medium before use. To study glucose toxicity on islets, MIN6 cells were incubated in DMEM medium with or without glucose at 12.5 mM and 25 mM concentrations for the indicated periods of time.

### Apoptosis assay

MIN6 cells, 5 × 10^5^ cells/well, were seeded to 6 well plates. The next day cells were transfected with SCR or INSR ASOs. 24 hours after transfection, medium was replaced with medium containing 0.1% serum with 1 μM Anisomycin or 0.5% (w/v) BSA with or without 0.5 mM palmitate and incubated at 37 °C for different periods of time. Next, the medium of each well was carefully transferred to a 15 ml tube cells were washed with PBS (which was also transferred into the appropriate tube), detached from the plate using 200 μl trypsin and collected to the same tube. Thereafter, the cells in the tubes were centrifuged for 5 min at 1500 rpm and supernatant was discarded. The cells were washed with 2 ml PBS, and cell pellet was resuspended with 50 μl Hanks balanced salt solution. Cell suspension was mixed with 0.4% Trypan blue in a 1:1 ratio and cells were counted using a BioRad cell counter (model TC-10). The remaining cells were centrifuged, lysed and subjected to western blotting to detect cleaved caspase-3.

### RT-PCR/Q-RT-PCR

Total RNA was extracted with Tri-reagent (Sigma) and 1 μg of total RNA was reverse transcribed using M-MLV reverse transcriptase (Promega). PCR was performed on1/10 volume (2 μl) of the cDNA, in 25 μl reactions containing 12.5 μl of PCR Mix (Kapa Biosystem), 1.25 μl of 10 μM forward and reverse Primer, 1.8 μl of DMSO. PCR conditions were as follows: 95 °C for 3 minutes, then 34 cycles of 95 °C for 15 sec, 60 °C for 15 sec, 72 °C for 45 sec followed by 10 minutes at 72 °C. PCR products were separated on 2% agarose gel. qPCR was performed on the cDNA using SYBR green (Roche) and CFX96 (Bio-rad) real time PCR machine. The PCR reaction is composed of the following steps: 95 °C for 10 minutes, 95 °C for 5 sec, 58 °C for 20 sec + plate read, back to step 2 39 times, Melt curve 60 °C to 95 °C, increment 0.5 °C for 5 sec + plate read. Results were analyzed with Bio-Rad CFX Manager software.

### Immunoblotting

Cells were lysed in Laemmli buffer and analyzed for total protein concentration. 20 μg of total protein from each cell lysate was separated by SDS-PAGE and transferred on to a PVDF membrane. The membranes were blocked with 5% milk and probed with specific antibodies. Bands were visualized using enhanced chemiluminescence detection. Primary antibodies: β-Catenin (1:2000, Sigma), Phospho-MEK S217/221 (1:1000, Cell Signaling), total MEK (1:1000, Cell Signaling), phospho-ERK T202/Y204 (1:1000 Sigma), total ERK1/2 (1:1000, Cell Signaling), phospho-AKT (1:1000, Cell Signaling), AKT (1:1000, Cell Signaling), cleaved Caspase 3 (1:1000, Cell Signaling) SRSF1 (AK96 culture supernatant 1:300), CUGBP1 (1:1000, Santa Cruz Biotechnology), MBNL1 (1:250, Santa Cruz Biotechnology), SRSF3 (1:1000, Santa Cruz Biotechnology). Secondary antibodies: HRP-conjugated goat anti-mouse, goat anti-rabbit and donkey anti-goat IgG (H+L; 1:10000 Jackson Laboratories).

### Statistical analysis

All the experiments described in this paper were repeated at least 3 times. Number of repeats is indicated in figure legends (n=). Significant P-values, calculated by two-tailed student T-test, are shown as (*) and the value appears in the figure legends.

## Results

### Insulin induces inclusion of *INSR* exon 11

**Figure 1:**
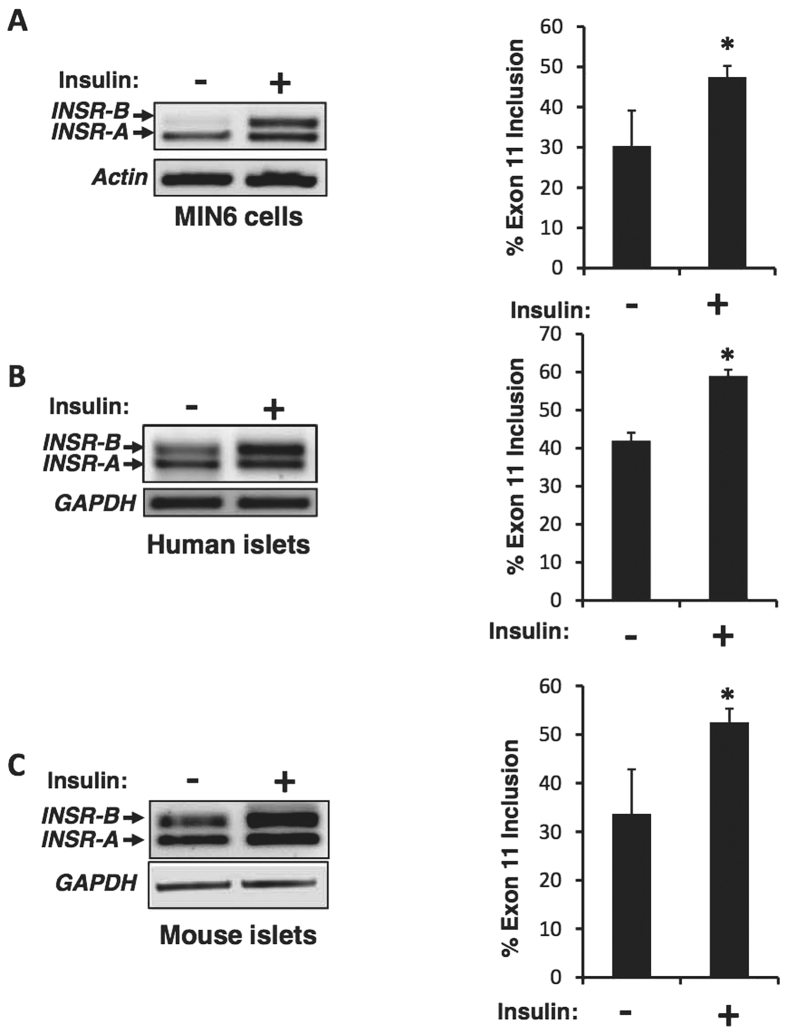
Insulin induces INSR exon 11 inclusion in pancreatic beta cells. (**A**) MIN6 cells were starved for 24 hours in 0.1% serum and then stimulated for 24 hours with 100 nM insulin. Total RNA was extracted and subjected to RT-PCR using primers from exon 10 and 12 of *INSR*. Actin was used as control. (**B**) Human Islets were stimulated with insulin and subjected to RT-PCR as in (**A**). GAPDH served as an RT-PCR control. (**C**) Mouse Islets were stimulated with insulin and subjected to RT-PCR as in (**A**). GAPDH served as an RT-PCR control. The left panels are representative gels and the right panels are quantification of % exon 11 inclusion from 3 individual experiments. Error bars, SD; n = 3, *p < 0.05.

Pancreatic beta cells secrete insulin in response to elevated blood glucose. Some of the secreted insulin binds and activates insulin receptors on the membranes of the beta cells in an autocrine manner^30^, and has a known pro-survival effect on beta cells. To examine whether signaling from the insulin receptor affects the alternative splicing of *INSR* pre-mRNA, we examined changes in *INSR* alternative splicing upon stimulation with insulin. Mouse insulinoma cell line (MIN6), isolated mouse pancreatic islets and human islets were starved for 24 hours in 0.1% serum and then stimulated for 24 hours with 100 nM insulin. Total RNA was extracted and subjected to RT-PCR and Q-RT-PCR. We found that insulin stimulation leads to increased exon 11 inclusion in both human islets, mouse islets and in the MIN6 cell line (Fig. 1, Fig. S1A,B).

### Insulin induces inclusion of *INSR* exon 11 through activation of the Ras-MAPK/ERK pathway

**Figure 2:**
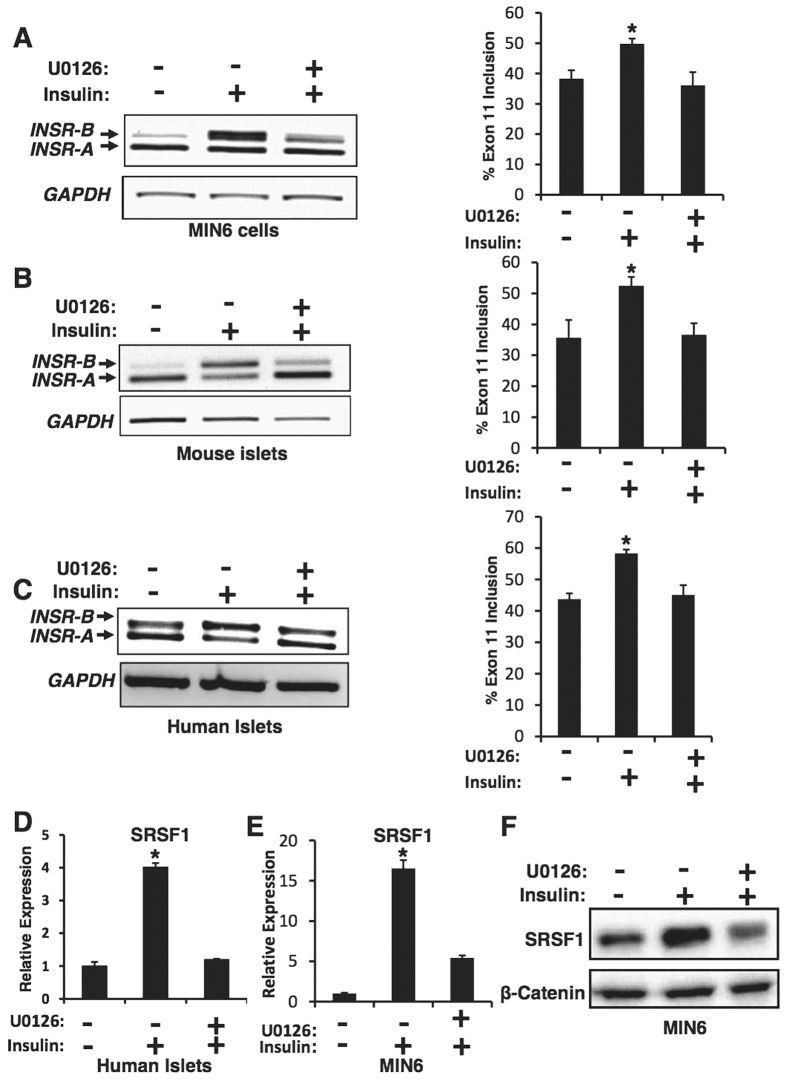
Insulin enhances SRSF1 expression and *INSR* exon 11 inclusion through the MAPK-ERK pathway. (**A**) MIN6 cells were starved for 24 hours in 0.1% serum and then stimulated with 100 nM insulin in the presence or absence of 10 μM U0126 or vehicle (0.025% DMSO). Total RNA was extracted and subjected to RT-PCR using primers from exon 10 and 12 of *INSR*. GAPDH was used as a control. (**B**,**C**) Mouse and human pancreatic islets were starved for 24 hours in 0.1% serum and then stimulated with 100 nM insulin for 24 hours, in the presence or absence of 10 μM U0126. RNA was extracted and *INSR* splicing detected as in (**A**). GAPDH served as a RT-PCR control. The right panels (**A**–**C**) are quantification of % exon 11 inclusion from 3 experiments. Error bars, SD; n = 3, *p < 0.05. (**D**) RNA was extracted from human islets described in (**C**) and subjected to qRT-PCR using primers specific for SRSF1. Tubulin was used for normalization. (**E**) RNA was extracted from MIN6 cells described in (**A**) and SRSF1 mRNA levels were detected by qRT-PCR using primers specific for SRSF1. Tubulin was used for normalization. (**F**) Western blot of protein lysates from cells described in (**E**).

Upon insulin binding, the insulin receptor autophosphorylates and then phosphorylates several substrates such as IRS-1 and IRS-2, which in turn bind adapter proteins and kinases and cause activation of the PI3K-Akt-mTOR and the Ras-Raf-MAPK pathways^30^. MAPK proteins play an important role in complex cellular programs like proliferation, differentiation, development, transformation and apoptosis^31^. In order to test whether the Ras-MAPK/ERK pathway mediates the effect of insulin on *INSR* alternative splicing, we pharmacologically blocked the Ras-MAPK/ERK pathway by the MEK1 inhibitor, U0126^32^ (Fig. S2). We stimulated pancreatic islets (human and murine) and MIN6 cells with insulin in the presence or absence of 10 μM U0126. We found that blocking the insulin-activated Ras-MAPK/ERK pathway by U0126 leads to inhibition of exon 11 inclusion in MIN6 cells, mouse and human pancreatic islets after treatment with insulin (Fig. 2A–C, Fig. S1A,B).

These results suggest that alternative splicing of the insulin receptor is regulated by signals emanating from the insulin receptor and are mediated by the Ras-MAPK-ERK signaling pathway.

### Insulin enhances the expression of SRSF1 through the Ras-MAPK/ERK pathway

Previous reports^26^ suggest that the splicing factor SRSF1 induces inclusion of *INSR* exon 11 by binding to exonic splicing enhancers in exon 11. We recently have shown that activated Ras, which activates the Raf-MEK-ERK pathway, elevates SRSF1 levels which in turn alter the splicing pattern of its targets^33^. Since activation of the Ras-MAPK/ERK pathway through the insulin receptor induces inclusion of exon 11 of the *INSR*, we sought to determine whether it regulates the expression of SRSF1 as well. MIN6 cells and human pancreatic islets were treated with insulin in the presence or absence of the MEK inhibitor, U0126. We determined SRSF1 transcript levels by Q-RT-PCR (Fig. 2D,E) and SRSF1 protein levels by Western blot (Fig. 2F). We found that insulin enhances the expression of SRSF1 and this effect was reversed by U0126. This suggests that insulin affects SRSF1 expression through the Ras-MAPK/ERK pathway, which in turn induces the alternative splicing observed in *INSR* exon 11. We further examined the expression of additional splicing factors known to regulate *INSR* exon 11 splicing^26^. We found that the levels of CELF1 (CUGBP1) did not change and the levels of SRSF3 (SRp20) were slightly increased. However, MBNL1 levels were significantly up-regulated upon insulin stimulation (Fig. S3A–D). These results suggest that MBNL1, in addition to SRSF1, contributes to *INSR* exon 11 inclusion upon insulin stimulation.

### Activation of glucose metabolism in beta cells *in vitro* and vivo induces *INSR* exon 11 inclusion

**Figure 3:**
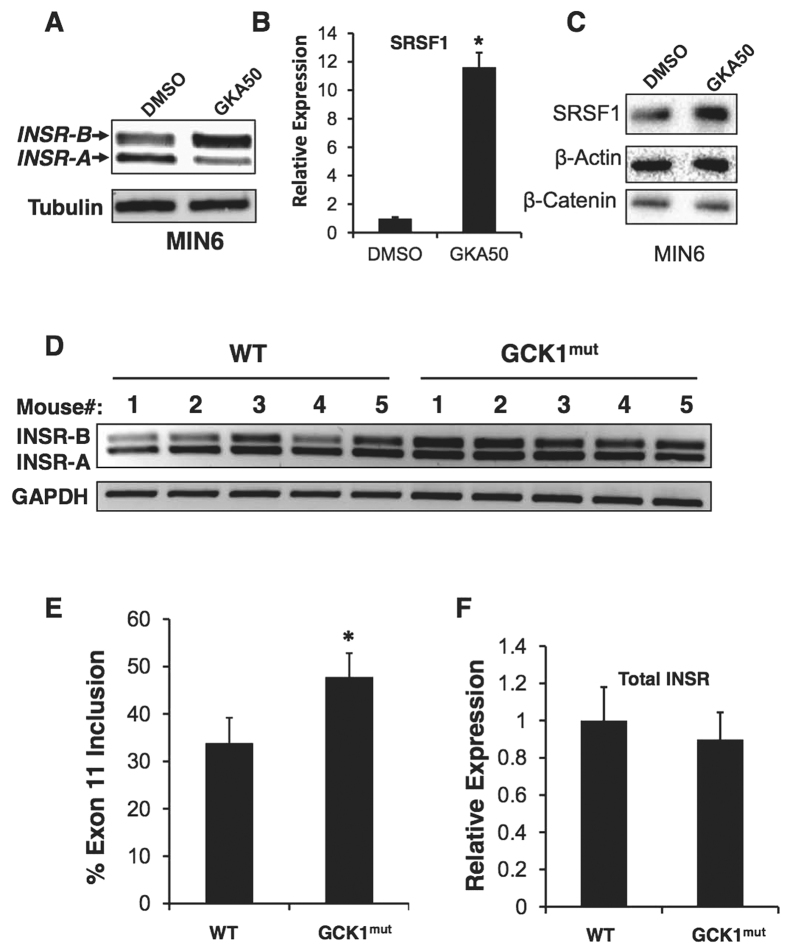
GCK1 activation upregulates SRSF1 and induces INSR exon 11 inclusion. (**A**) MIN6 cells were exposed to DMEM medium without glucose containing DMSO with or without 25 μM GKA50 for 48 hours. Total RNA was extracted and subjected to RT-PCR using primers from exon 10 and 12 of *INSR*. Tubulin was used as control. (**B**) Total SRSF1 mRNA levels from cells described in (**A**) were detected by qRT-PCR using primers from exon 2 and 3.Tubulin was used for normalization. (**C**) Western blot of lysates from cells described in (**A**). (**D**) Total RNA was isolated from mouse islets of control mice (n = 5) or mice overexpressing GCK1 specifically in β-cells (n = 5) and subjected to RT-PCR using primers from exon 10 and 12 of *INSR*. GAPDH served as an RT-PCR control. (**E**) Graph showing quantitation of % exon 11 inclusion in (**D**). (**F**) Q-RT-PCR of insulin expression in samples described in (**D**).

Increased blood glucose levels activate glucokinase in beta cells, which in turn initiates glucose metabolism into ATP, leading to opening of K-ATP channels, depolarization and eventually insulin secretion^2^,^3^. To examine if glucokinase activation leads to increased inclusion of *INSR* exon 11, we activated glucokinase *in vitro* in MIN6 cells by addition of the glucokinase agonist GKA50^34^. We observed that glucokinase activation increased *INSR* exon 11 inclusion (Fig. 3A, Fig. S1C), as well as enhanced SRSF1 expression (Fig. 3B,C). To examine if elevated glucokinase activity *in vivo* (which leads to elevated secretion of insulin from beta cells *in vivo*) leads to inclusion of *INSR* exon 11, we took advantage of a transgenic mouse model conditionally expressing a constitutively active mutant of glucokinase (GCK) in beta-cells^29^. Beta cells from GCK1 mutant mice show enhanced proliferation initially after transgene expression, and subsequently DNA damage accumulates, leading to beta cell apoptosis^29^. Islets from wild type and GCK1 mutant mice were isolated after transgene induction at a time point where beta cells undergo proliferation. RNA isolated from the pancreatic islets of these mice was compared with islets from wild type mice. Most of the mice expressing the active mutant of glucokinase-1 showed increased inclusion of *INSR* exon 11, suggesting that elevated glucose metabolism (which leads to elevated insulin secretion) enhances exon 11 inclusion *in vivo* in mice (Fig. 3D–F).

### SRSF1 mediates *INSR* exon 11 inclusion upon insulin stimulation

**Figure 4:**
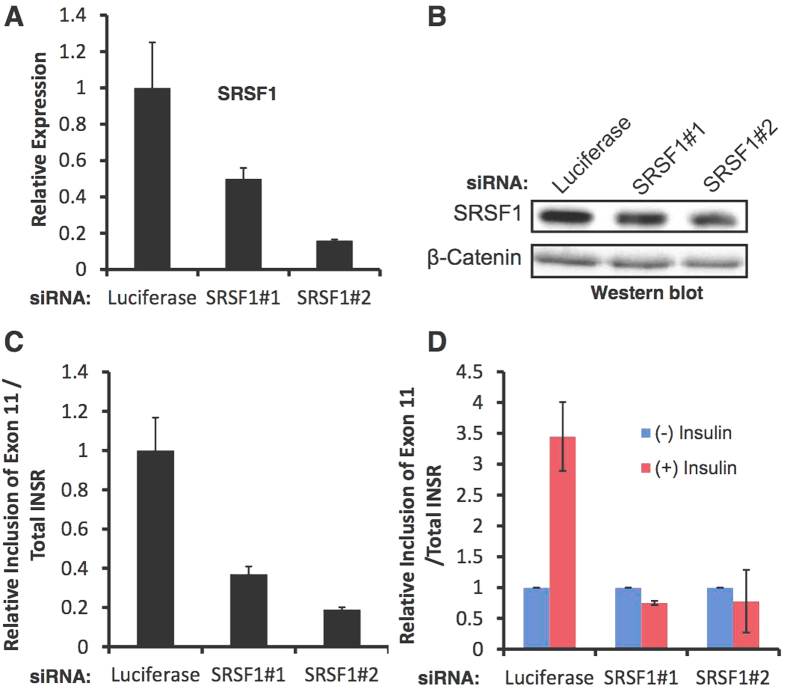
SRSF1 knockdown blocks INSR exon 11 inclusion upon insulin stimulation. (**A**) MIN6 cells were transfected with the indicated siRNAs for 96 hours. RNA was extracted and SRSF1 levels were detected by Q-RT-PCR. (**B**) Cells described in (**A**) were lysed and SRSF1 protein levels were detected by Westen blot. β-catenin was used as loading control. (**C**) INSR exon 11 inclusion was detected in RNA from cells described in (**A**) by Q-RT-PCR. (**D**) MIN6 cells were transfected with the indicated siRNAs for 36 hours, starved in low serum over night, and induced with insulin for 24 hours. INSR exon 11 inclusion was detected by Q-RT-PCR. Error bars show SD; n = 3.

In order to examine if SRSF1 up-regulation upon insulin stimulation is causatively involved in the inclusion of *INSR* exon 11, we stimulated MIN6 cells with insulin after transfection with SRSF1 siRNAs or a control siRNA (Fig. 4A,B). SRSF1 knockdown reduced *INSR* exon 11 inclusion under normal serum conditions (Fig. 4C), and blocked *INSR* exon 11 inclusion upon stimulation with insulin (Fig. 4D). These results suggest that SRSF1 mediates *INSR* exon 11 inclusion upon insulin stimulation.

### Toxic glucose and lipid levels induce *INSR* exon 11 skipping in MIN6 cells

**Figure 5:**
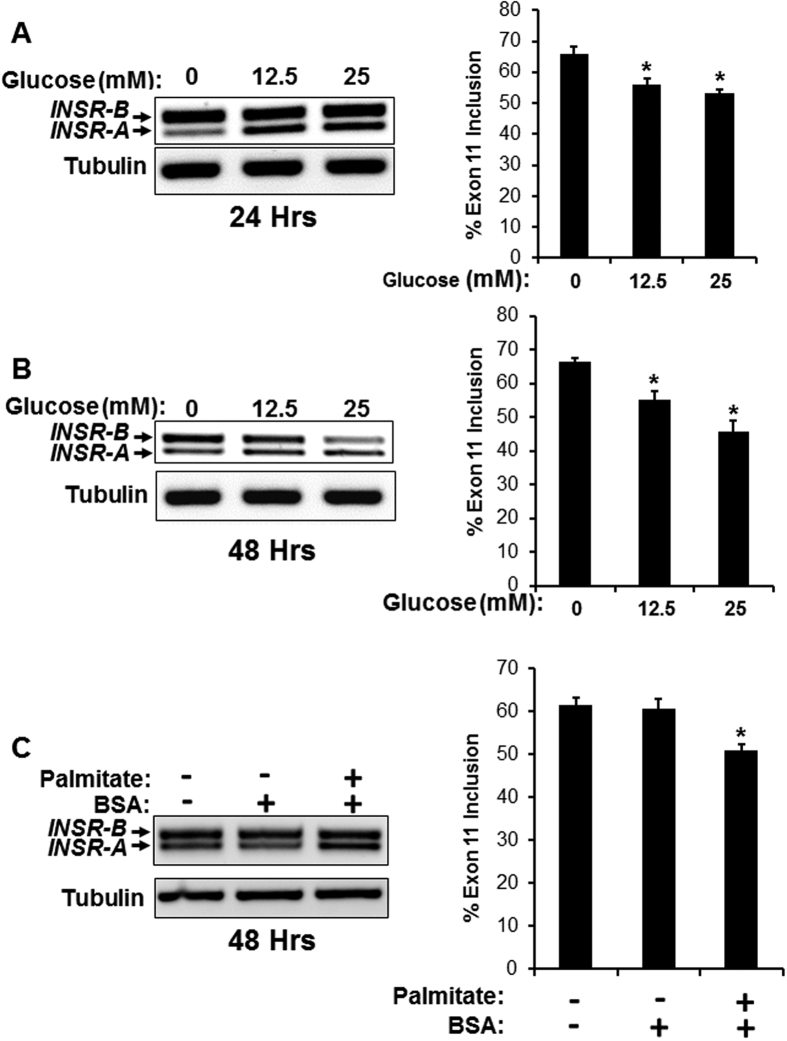
High glucose and lipid levels induce *INSR* exon 11 skipping in MIN6 cells. (**A**,**B**) MIN6 cells were incubated in glucose-free DMEM medium. After 24 hours medium was replaced with DMEM medium without or with 12.5 mM or 25 mM glucose for 24 and 48 hours. After stipulated period of time, RNA was isolated and subjected to RT-PCR to detect the splicing of *INSR*. Tubulin served as a RT-PCR control. (**C**) MIN6 cells were incubated in normal MIN6 medium with 0.5% (w/v) BSA with or without 0.5 mM palmitate for 48 hours. After stimulation, RNA was isolated and subjected to RT-PCR to detect the splicing of *INSR*. Tubulin served as a RT-PCR control. The right panels represent quantification of % exon 11 inclusion from 3 individual experiments. Error bars, SD; n = 3, *p < 0.05.

High glucose and lipid blood levels are hallmarks of T2DM and are known to induce beta cell toxicity and apoptosis^35^. To examine if high levels of glucose or lipids (which induce toxicity) act differently on *INSR* splicing than enhanced glucose metabolism by glucokinase (which leads to elevated insulin secretion), we exposed MIN6 cells to high levels of glucose or palmitate. High glucose or palmitate levels induced skipping of *INSR* exon 11 (Fig. 5). These results suggest that, unlike activation of glucokinase (which leads to increased glucose metabolism and eventually to insulin secretion), toxic glucose and lipid levels induce the opposite effect on *INSR* alternative splicing. No change in SRSF1 expression was detected in high glucose conditions (Fig. S4A,B). Examining the levels of additional splicing factors involved in *INSR* splicing regulation revealed that while protein levels of CELF1, SRSF3 and SRSF1 did not change, the levels of MBNL1, which is known to enhance *INSR* exon 11 inclusion^16^ decreased in high glucose conditions (Fig. S4B,C). This result points to a specific role for MBNL1 reduction in mediating *INSR* exon 11 skipping under high glucose levels.

### A high INSR-A/INSR-B ratio sensitizes β-cells to stress-induced apoptosis

**Figure 6:**
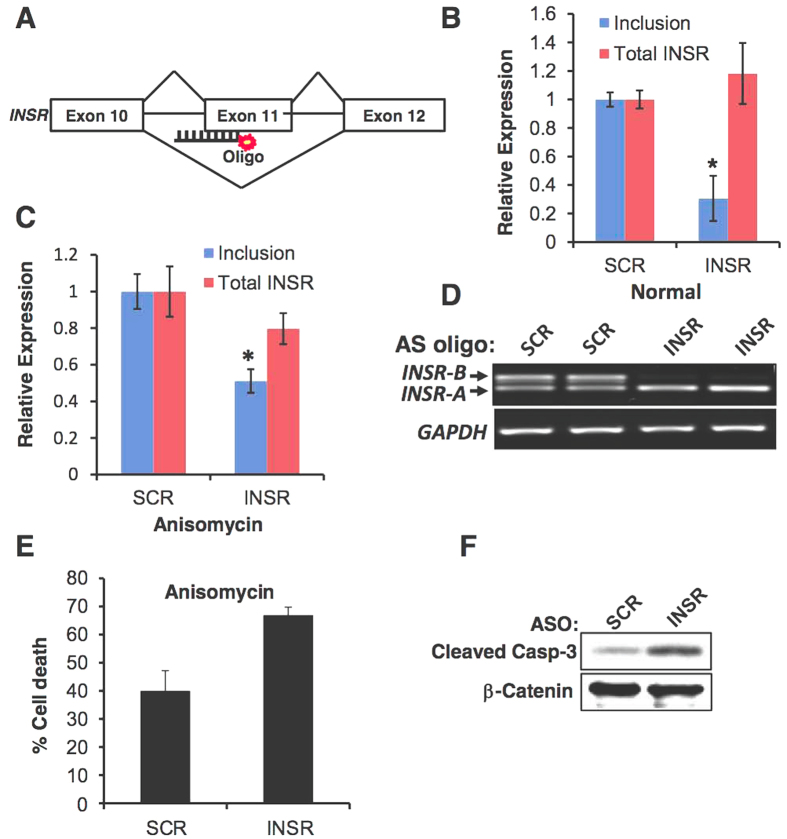
Induction of *INSR* exon 11 skipping sensitized MIN6 cells to stress-induced apoptosis. (**A**) A scheme showing the position of the Cy5 labeled 2′-O-Met antisense RNA oligo on *INSR* intron 10-exon 11 junction. (**B**) MIN6 cells were transfected with 2.5 μM INSR specific or scrambled RNA oligos. 48 h after transfection total RNA was extracted and subjected to Q-RT-PCR. (**C**) Cells described in (**B**) were treated with 1 μM anisomycin for 48 hours. RNA was extracted and subjected to Q-RT-PCR. (**D**) RT-PCR of RNA from cells described in (**B**). (**E**) Cells described in (**C**) were analyzed by trypan blue exclusion assay to determine % cell death (**F**). Cells described in (**C**) were analyzed by western blot. Cleaved caspase-3 served as a marker for apoptosis. β-catenin was used as a loading control.

In order to understand the role of insulin receptor alternative splicing change in pancreatic beta cell survival we designed a 2′-O-Methyl- antisense RNA oligonucleotide (ASO) that competes with *INSR* intron 10 3′-splice site to induce exon 11 skipping. (Figure 6A). Introduction of the antisense oligo into MIN6 cells induced efficient skipping of *INSR* exon 11 (Fig. 6B–D). Scrambled (SCR) RNA oligonucleotide was used as a control. In order to examine the effects of insulin receptor splicing variants on beta cells proliferation and survival, we directly manipulated the splicing of *INSR* by the RNA ASOs. MIN6 cells were transfected with either INSR or SCR ASOs and then exposed to stress by the addition of anisomycin. Anisomycin is an antibiotic which inhibits protein synthesis by inhibiting peptidyl transferase of the 80S ribosome system, and is widely used to assay stress-induced apoptosis^36^. Cells transfected with INSR ASO induced skipping of exon 11 (Fig. 6C) and showed a 1.5–2 fold increase in cell death upon exposure to anisomycin compared to the SCR transfected controls (Fig. 6E). Anisomycin induced cell death was due to induction of apoptosis as detected by caspase-3 cleavage (Fig. 6F). This result suggests that modulation of *INSR* splicing towards INSR-A sensitizes β-cells to stress-induced apoptosis.

### A high INSR-A/INSR-B ratio sensitizes β-cells to lipotoxicity

**Figure 7:**
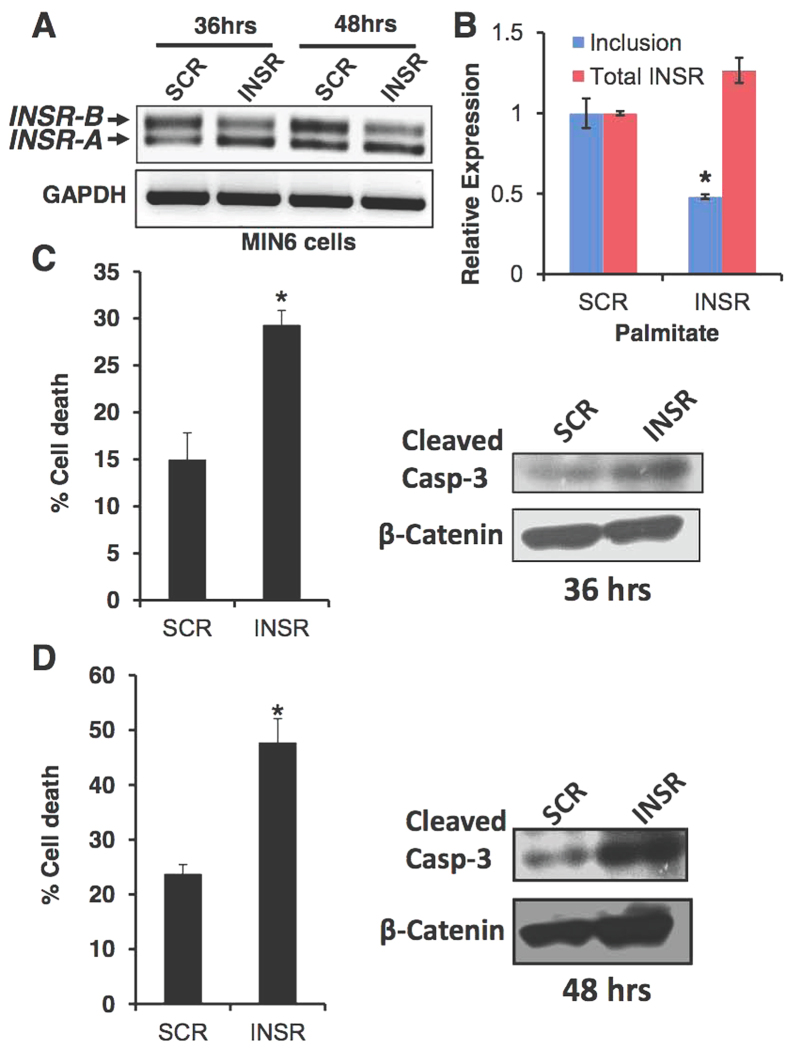
Induction of *INSR* exon 11 skipping sensitized MIN6 cells to lipotoxicity-induced apoptosis. (**A**) MIN6 cells were transfected with 2.5 μM INSR specific or scrambled RNA oligos. 24 h after transfection medium was replaced with medium containing 0.1% serum with 0.5% (w/v) BSA with or without 0.5 mM palmitate for 36 or 48 hrs. Total RNA was extracted and subjected to RT-PCR using primers from exon 10 to 12 of *INSR*. (**B**) Q-RT-PCR of cells described in (**A**). (**C**,**D)** Left panels. Cell death in cells described in (**A**) was measured by trypan blue exclusion assay. Right panels: Western blot of the cells described in (**A**) probed with antibodies to cleaved Caspase -3. β-catenin was used as a loading control.

Lipotoxicity is one of the physiological stresses that lead to beta cell dysfunction, apoptosis and the development of T2DM^35^. We examined the effects of high glucose in combination with a high concentration of fatty acids (palmitate) on MIN6 survival with or without induction of *INSR* exon 11 skipping. Induction of *INSR* exon 11 skipping by ASO, followed by treatment with high palmitate concentrations for 36 or 48 hours (Fig. 7A,B) enhanced apoptosis of MIN6 cells in response to gluco-lipotoxicity conditions, as measured by trypan blue exclusion assay and caspase 3 cleavage (Fig. 7C,D). Thus, skipping of *INSR* exon 11 enhances the sensitivity of MIN6 cells to high palmitate concentration. Taken together, these results suggest that the INSR-B isoform is protective and skipping of exon 11 sensitizes beta cells to high fatty acid stress conditions.

### Induction of alternative splicing in *INSR* inhibits the MEK1-ERK pathway

**Figure 8:**
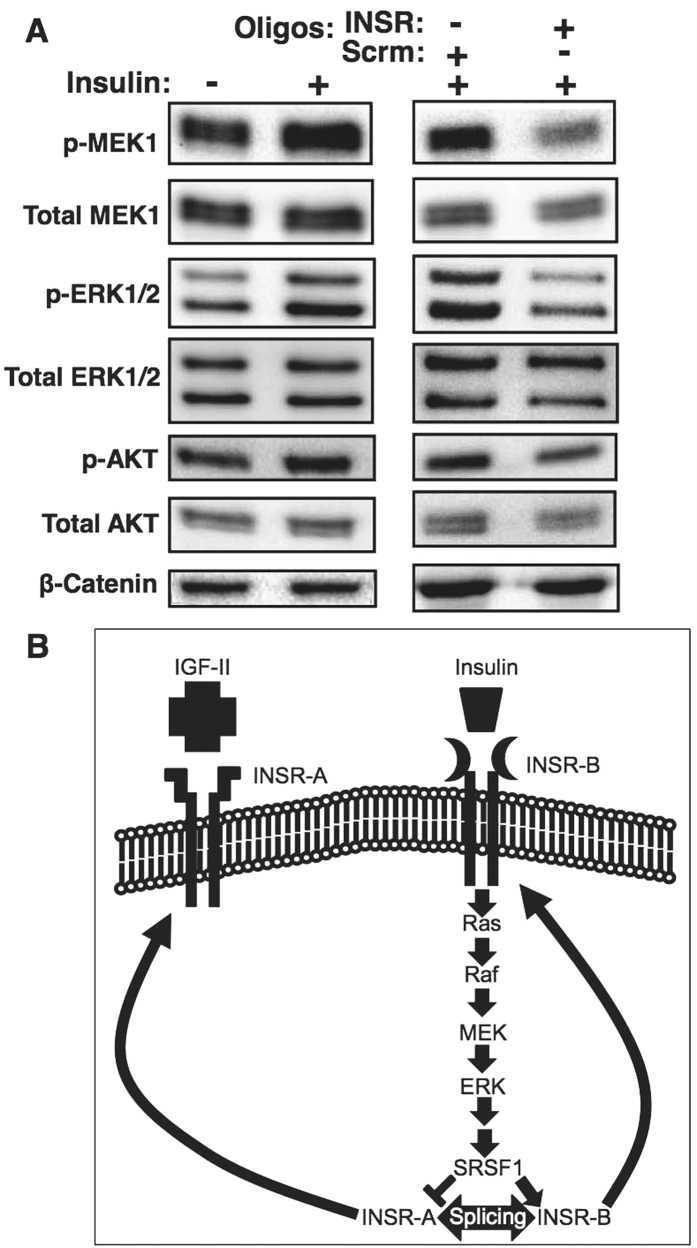
Induction of *INSR* exon 11 skipping inhibits insulin–induced activation of the Ras-MAPK-ERK pathway. (**A**) (Left): Western blot of MIN6 cells starved for 24 hours in 0.1% serum and then stimulated with 100 nM insulin for 1 hour. β-catenin was used as loading control. (Right): Western blot of MIN6 cells transfected with either 2.5 μM *INSR* specific or scrambled RNA oligos, serum starved, and then stimulated for 1 hour with insulin. β-catenin was used as loading control. (**B**) Schematic representation of the regulation of *INSR* splicing by insulin signaling through the MAPK-ERK pathway.

INSR transmits signals to activate both the PI3K-mTOR and the Ras-MAPK pathways^37^. To understand whether induction of *INSR* exon 11 skipping alters downstream signaling in beta cells, we examined the activity of these pathways after treatment with the INSR ASOs. We found that skipping of *INSR* exon 11 in MIN6 cells reduced MEK1 and ERK phosphorylation, as well as phosphorylation of Akt indicating that the Ras-MAPK/ERK and PI3K-mTOR pathways were inhibited (Fig. 8A). These results suggest that in beta cells INSR-B is a stronger driver of insulin downstream signal transduction than INSR-A, which might explain its protective properties.

## Discussion

The insulin receptor is essential for pancreatic beta cell function and survival^4^,^5^,^6^, and insulin signaling is a key signaling pathway in beta cell survival^7^,^8^,^38^. *INSR* alternative splicing is perturbed in several disease conditions^18^,^19^, and the INSR-A isoform may play an important role in cancer development^18^. However, the role of *INSR* alternative splicing in pancreatic beta cell function and survival is currently unknown.

In this study we found that insulin signaling, through activation of the Ras-MAPK pathway, up-regulates the splicing factor SRSF1 and induces inclusion of *INSR* exon 11 to generate elevated levels of the INSR-B isoform (Figs 1 and 2). Blocking MEK1-ERK signaling pharmacologically inhibits insulin induction of SRSF1 and inclusion of *INSR* exon 11 (Fig. 2). SRSF1 is a known regulator of *INSR* exon 11 inclusion^39^. However, other splicing factors might also play a role in regulation of *INSR* splicing in response to insulin^12^,^16^,^40^. Indeed, our data shows that, similarly to SRSF1, MBNL1 is also up-regulated upon insulin stimulation and this up-regulation is blocked by MEK1 inhibition (Fig. S3). Taken together, these results suggest that both SRSF1 and MBNL1 enhance *INSR* exon 11 inclusion upon insulin stimulation.

The basal levels of *INSR* exon 11 inclusion are affected by environmental conditions. While low serum conditions (prior to insulin stimulation) encourage exon 11 skipping (Figs 1 and 2), low glucose conditions (prior to glucose/palmitate stimulation) encourage exon 11 inclusion (Figs 5 and 7). These effects can be mediated by cell density as well as signaling from growth factors and glucose metabolism.

Although it is now clear that activation of the INSR and components of its downstream signaling pathway are important for beta cell function and survival^4^,^38^,^41^,^42^, it is not clear how *INSR* alternative splicing is involved in beta cell function or survival. In hepatocellular carcinoma and other cancer cells, the INSR-A splicing isoform (which lacks exon 11) is up-regulated and promotes cancer development and maintenance^13^,^18^,^43^. The INSR-A isoform can bind IGF-II, in addition to insulin, unlike the INSR-B isoform which can only bind insulin with high affinity^14^,^43^. It is thus possible that, in beta cells, the autocrine secretion of insulin is more important for survival and proliferation than IGF-II signaling. While IGF-II signaling might be more important in other tissues, such as liver and thyroid, thus leading to malignant transformation when the INSR-A isoform is overexpressed^13^,^14^,^18^,^43^. To examine if enhanced glucose metabolism in beta cells (which leads to elevated insulin secretion) also induces *INSR* exon 11 inclusion we activated glucokinase (GCK) *in vitro*, with the glucokinase agonist GKA50, and *in vivo*, using beta cells of transgenic mice harboring a constitutively active mutant of GCK^29^. We found that indeed GCK activation, both *in vitro* and *in vivo*, increased inclusion of *INSR* exon 11 in beta cells (Fig. 3). These findings suggest that increased glucose metabolism and insulin secretion lead to exon 11 inclusion *in vivo* (Fig. 3). An alternative splicing switch that reduces INSR-B and elevates INSR-A also occurs in myotonic dystrophy type 1 and 2 (DM1, DM2) and correlates with increased insulin resistance and diabetes^12^,^17^. This splicing switch, which occurs because of toxic repetitive RNA triplets that alter the activity of splicing factors from the CELF and MBNL families^16^,^40^,^44^, affects insulin resistance of the peripheral tissues but might affect beta cell survival and function^12^,^17^,^45^. In order to demonstrate a causative link of SRSF1 up-regulation following insulin stimulation to *INSR* exon 11 inclusion, we examined if SRSF1 knockdown can prevent *INSR* exon 11 inclusion induced by insulin. SRSF1 knockdown reduced *INSR* exon 11 inclusion in MIN6 cells under normal serum condition and completely abolished *INSR* exon 11 inclusion following insulin stimulation, suggesting that indeed SRSF1 mediates insulin-induced *INSR* exon 11 inclusion (Fig. 4). We further found that MBNL1 is also up-regulated by insulin (Fig. S3) and thus it is likely that both factors contribute to insulin-induced *INSR* exon 11 inclusion. While insulin is a pro-survival factor for beta cells, high glucose and lipid levels in the blood are toxic to beta cells and mediate beta cell loss in type 2 diabetes^2^. To examine the effects of high glucose and lipid levels on INSR alternative splicing we exposed MIN6 cells to high glucose or palmitate levels, and found that it increased *INSR* exon 11 skipping (Fig. 5). These results imply that, by modulating *INSR* splicing, high glucose and lipid levels affect downstream signaling from the insulin receptor which might enhance, for example, insulin resistance, which is a very common phenomena seen in early stages of T2DM^2^. Interestingly, only MBNL1 protein levels were decreased at high glucose levels while SRSF1, CELF1 and SRSF3 levels did not change (Fig. S4). This suggests that toxic high glucose levels affect the regulation of INSR alternative splicing differently than insulin. Further investigation is needed to examine if MBNL1 levels are decreased in beta cells of diabetic mice and humans and how MBNL1 levels are connected to beta cell survival in diabetes. To study the role of *INSR* splicing on beta cell survival directly, we manipulated skipping of exon 11 by modified antisense RNA oligos that interfere with intron 10-exon 11 splice site. Introduction of these oligos in MIN6 cells result in the concomitant reduction of INSR-B and elevation of INSR-A levels in MIN6 cells, (Fig. 6A–D). Induction of exon 11 skipping sensitized the cells to stress-induced apoptosis (Fig. 6). Similar results were obtained with a more physiological toxic stimulus of gluco-lipotoxicity (Fig. 7). These results suggest that in beta cells, INSR-B, which is responsive to insulin but not to IGF-II is important for survival. A recent study showed that in patients recovering from diabetes after bariatric surgery, INSR-B levels (in visceral fat) correlated with weight loss and negatively correlated with blood insulin levels^19^. These results suggest that there is a tight connection between *INSR* splicing in fat tissue and blood glucose and insulin levels. Nevertheless, it is not clear what type of correlation exists under the same conditions in pancreatic beta cells.

In order to understand if *INSR* alternative splicing affects downstream signaling in beta cells we examined the activity of the Ras-MAPK-ERK and PI3K-mTOR pathways upon modulation of *INSR* alternative splicing by ASOs. We found that forcing *INSR* exon 11 skipping by ASOs inhibited phosphorylation of MEK1, ERK1/2 and Akt, suggesting that in MIN6 cells INSR-A is a weaker activator of these pathways downstream to insulin (Fig. 8). Because both the MEK1-ERK and Akt-mTOR pathways are pro-survival signaling pathways, it is possible that since INSR-B is a stronger activator of these pathways downstream to insulin in beta cells, elevated inclusion of exon 11 promotes beta cell survival in a positive feedback manner (Fig. 8B).

In conclusion, we found a new survival pathway in pancreatic beta cells that emanates from the insulin receptor, through activation of the Ras-MAPK pathway, leading to SRSF1 elevation and increased production of the INSR-B splicing isoform. We further show that INSR-B protects beta cells from stress-induced cell death. A direct clinical application of this finding would be to increase INSR-B levels in beta cells in order to increase their protection from diabetes related insults such as gluco-lipotoxicity.

## Additional Information

**How to cite this article**: Malakar, P. *et al*. Insulin receptor alternative splicing is regulated by insulin signaling and modulates beta cell survival. *Sci. Rep.* **6**, 31222; doi: 10.1038/srep31222 (2016).

## Supplementary Material

Supplementary Information
