# Supplementary material for: Insulin receptor alternative splicing is regulated by insulin signaling and modulates beta cell survival
Source: Sci Rep. 2016 Aug 16;6:31222. doi: 10.1038/srep31222 (PMC4985653; doi:10.1038/srep31222)
Supplement: Supplementary Information [file srep31222-s1.pdf]

Insulin receptor alternative splicing is regulated by insulin signaling and modulates beta cell survival

Pushkar Malakar[1,4], Lital Chartarifsky[1,4], Ayat Hija[2], Gil Leibowitz [3], Benjamin Glaser [3], Yuval Dor[2], and Rotem Karni[1]*

Figures S1-S5, Figure Legends, Table S1– List of primers used in the study

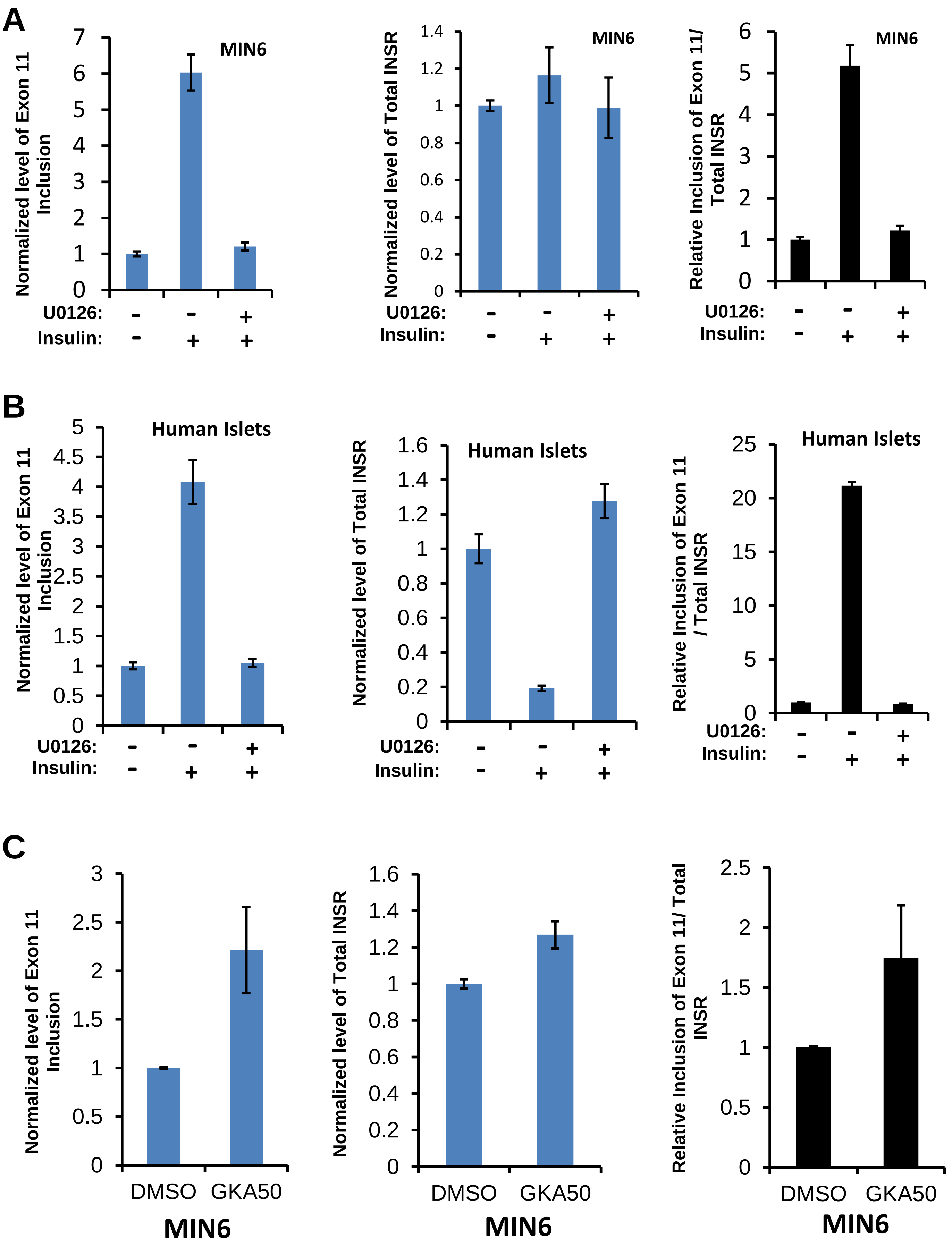

Malakar et al. Figure S1

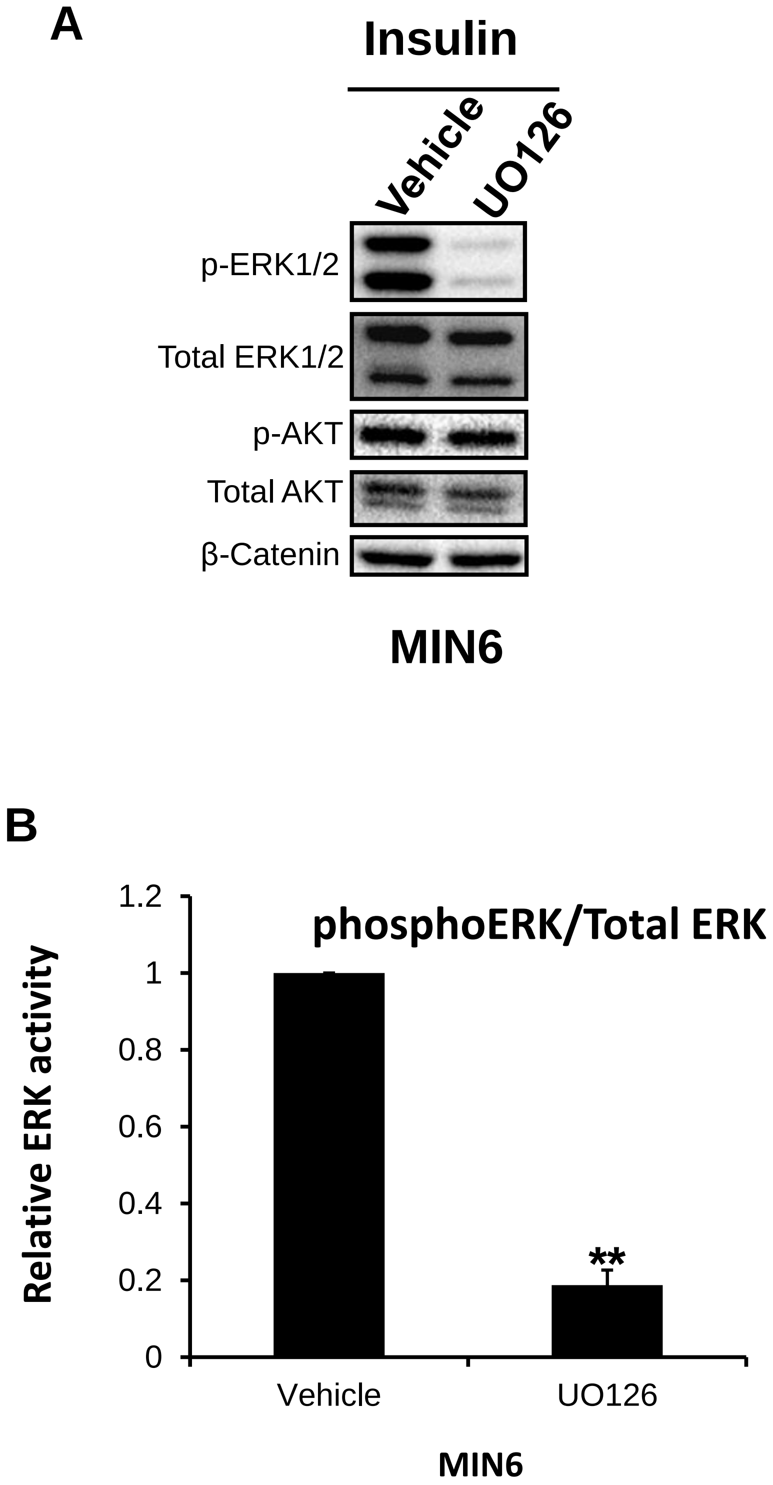

Malakar et al. Figure S2

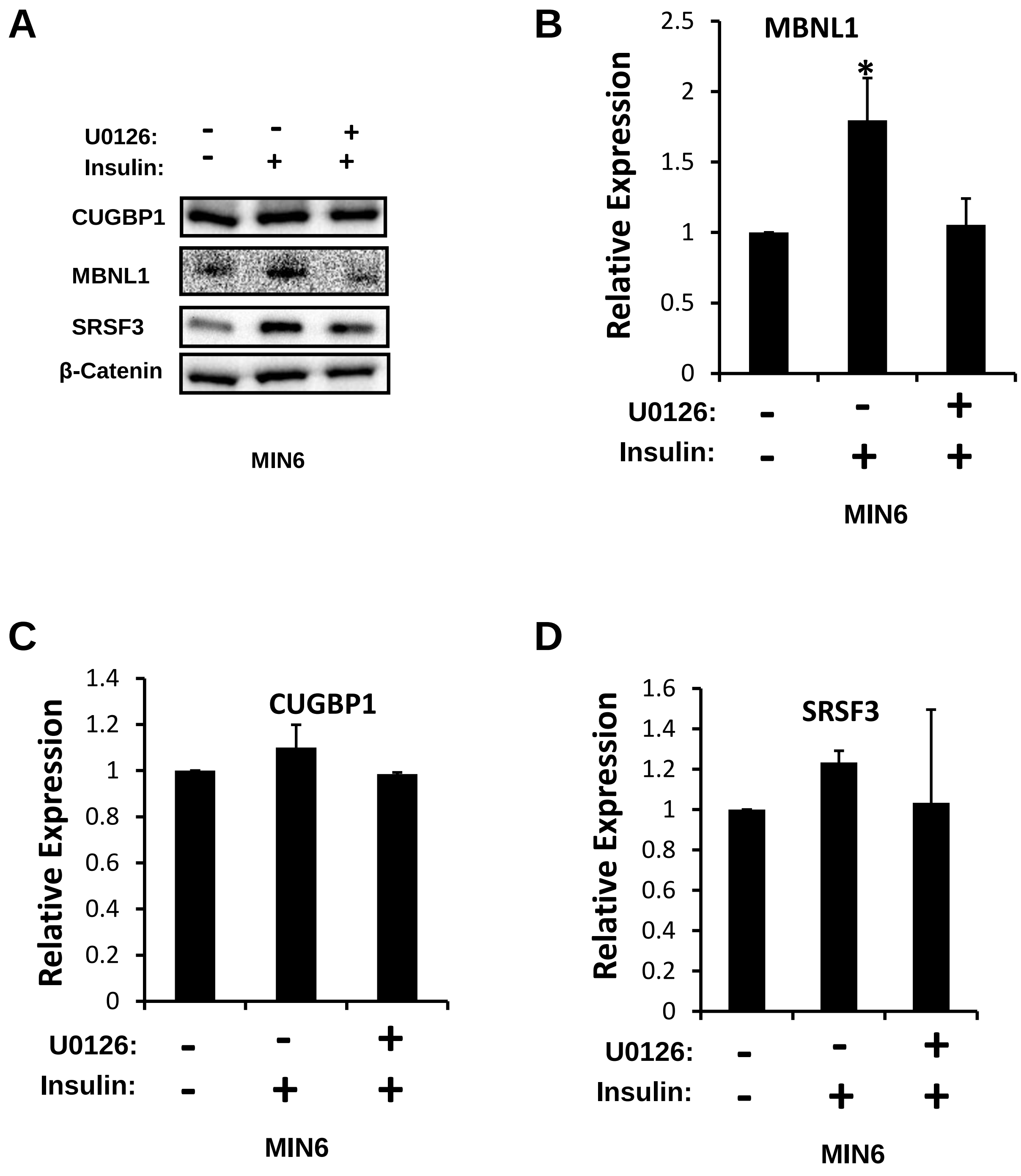

Malakar et al. Figure S3

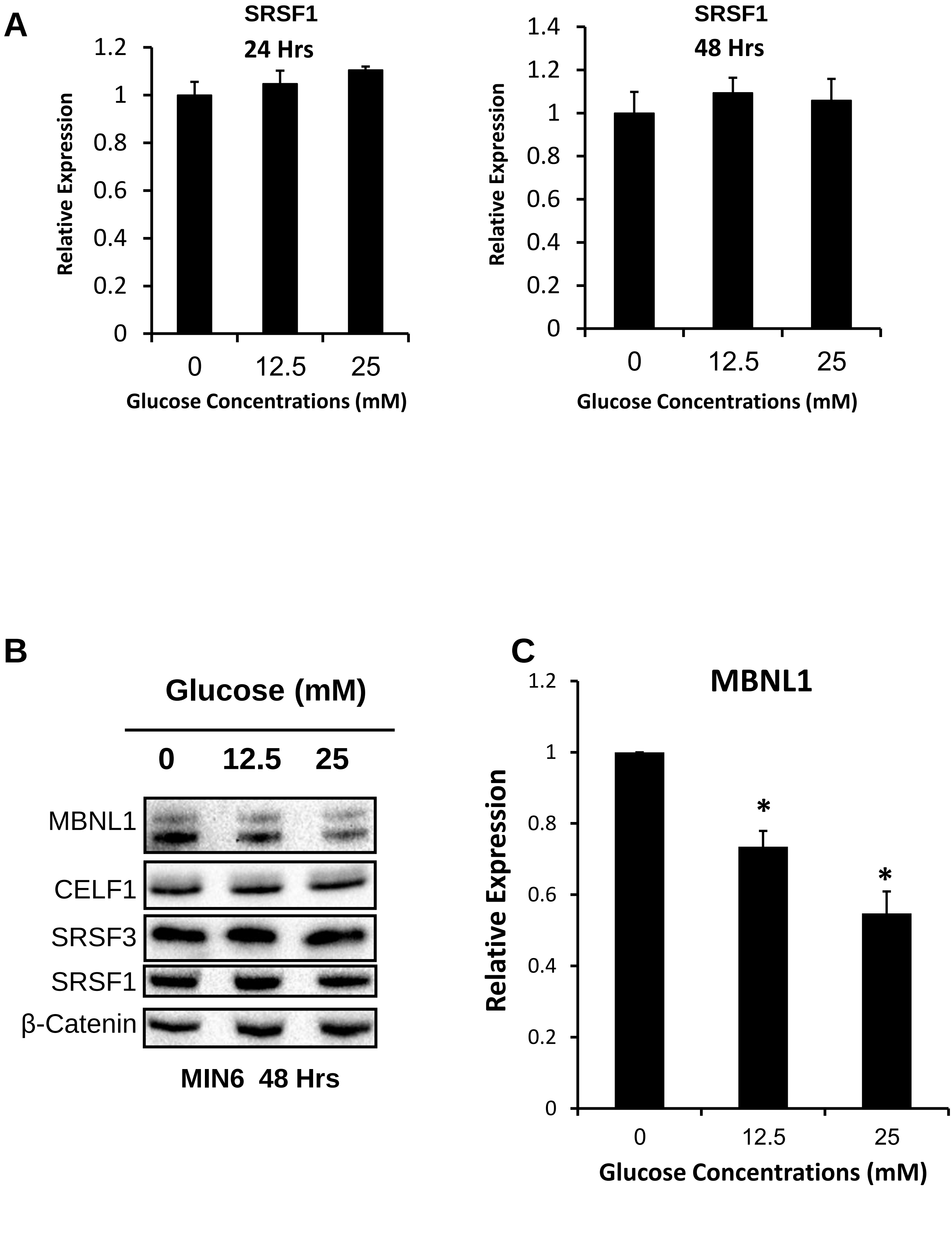

Malakar et al. Figure S4

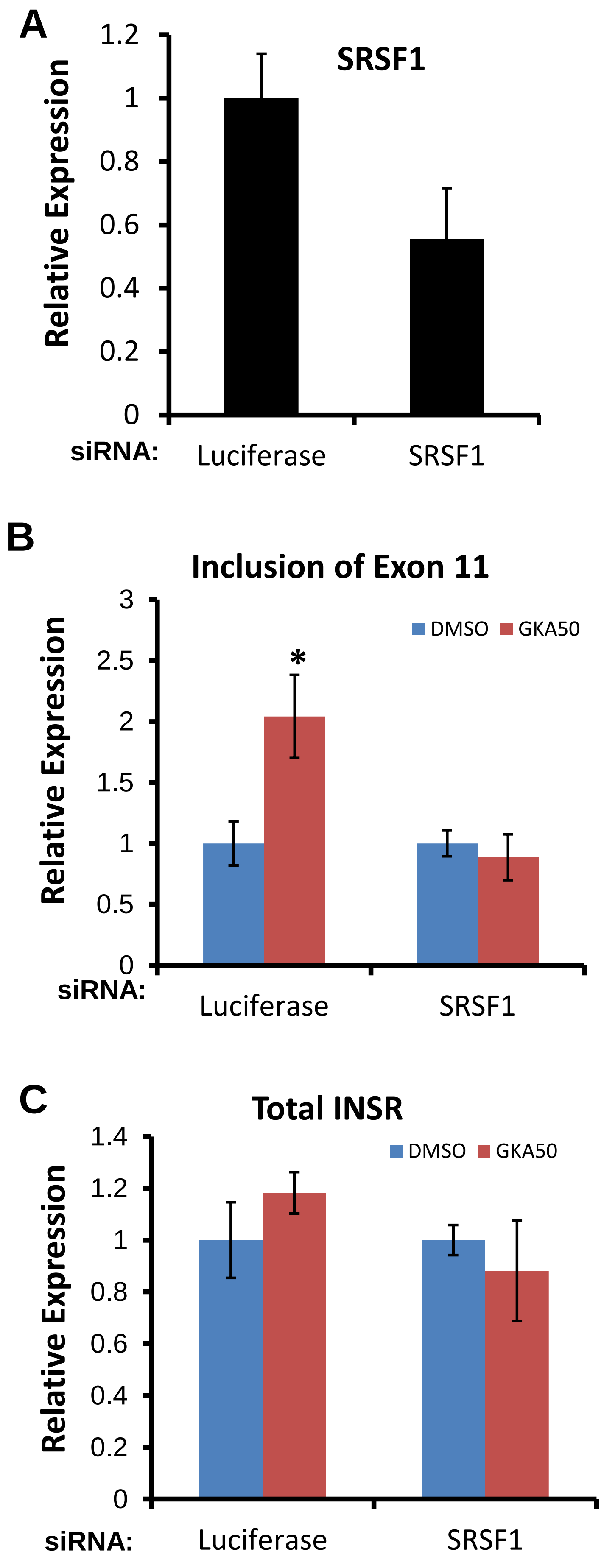

Malakar et al. Figure S5

Figure Legends:

Figure S1: Inclusion of *INSR* exon 11 by insulin and GCK1 activation through the MAPK-ERK pathway.

A: MIN6 cells were starved for 24 hours in 0.1% serum and then stimulated with 100nM insulin in the presence or absence of 10µM U0126. Total RNA was extracted and subjected to Q-RT-PCR using primers from exon 10 and exon 11, for inclusion and exon 1 and exon 2, for total INSR. Actin was used for normalization. *B*: Human pancreatic islets were starved for 24 hours in 0.1% serum and then stimulated with 100nM insulin in the presence or absence of 10µM U0126. RNA was extracted and the level of exon 11 inclusion of and total INSR was measured using Q-RT-PCR. Actin was used for normalization. *C*: MIN6 cells were grown in regular medium for 24 hours and then the medium was replaced with DMEM medium without glucose containing DMSO with or without 25 µM GKA50 for 48 hours. RNA was isolated and Q-RT-PCR performed to measure the exon 11 inclusion, total INSR and the relative inclusion of exon 11 as compared to total INSR.

Figure S2: Inhibition of ERK activity by UO126.

A: MIN6 cells were starved for 24 hours in 0.1% serum and then stimulated with 100nM insulin in presence or absence of 10µM U0126. Protein lysates were prepared and analyzed by western blot for ERK activity. β-Catenin was used as a loading control. *B*: Quantification of ERK activity in cells described in (A) as measured by phosphoERK/totalERK levels Error Bars, SD; n=3,**P<0.01.

Figure S3: Analysis of RNA binding proteins for their involvement in INSR splicing.

A: MIN6 cells were starved for 24 hours in 0.1% serum and then stimulated with 100nM insulin in the presence or absence of 10µM U0126. Western blot of lysates. β-Catenin was used as a loading control *B-D*: Quantification of the expression levels of MBNL1 (B), CUGBP1(C) and SRSF3 (D). Error Bars, SD; n=3, *P<0.05.

Figure S4: Analysis of RNA binding proteins for involvement in glucose induced toxicity leading to exclusion of *INSR* exon 11.

A: MIN6 cells were exposed to DMEM medium with or without 12.5mM or 25mM glucose for 24 and 48 hours. After stipulated period of time, RNA was isolated and subjected to Q-RT-PCR to

detect the expression of SRSF1. Tubulin was used for normalization. *B*: Western blot of lysates of MIN6 cells incubated with DMEM medium without or with 12.5mM or 25mM glucose for 48 hours. β-Catenin was used for normalization. *C*: Quantification of MBNL1 expression from Western blot shown in (B). Error Bars, SD; n=3, *P<0.05.

Figure S5: SRSF1 is important for the inclusion of *INSR* exon 11 through GCK1 activity.
A: MIN6 cells transfected with siRNA targeted against SRSF1 were grown for 24 hours and then the medium was replaced with DMEM medium without glucose containing DMSO with or without 25µM GKA50 for 48 hours. Cells were lysed, RNA and protein were isolated. Knockdown of SRSF1 was measured by Q-RT-PCR. Actin was used for normalization. *B*: Q-RT-PCR to detect exon 11 of INSR with (red) or without (blue) GCK1 activation by GKA50 in cells with either control siRNA or SRSF1 siRNA. Actin was used for normalization. *C*: Quantification of total INSR transcript levels (exons 1-2) measured by Q-RT-PCR Actin was used for normalization. Error Bars, SD; n=3,*P<0.01.

Primers

h = human, m = mouse

Semi-quantitative PCR

INSR E10 Forward AGATCCTGAAGGAGCTGGAGGA

hE12 Reverse GGTCGAGGAAGTGTTGGG

mE12 Reverse GAGGAGACGTTGGGGAAATCTG

GAPDH Forward ATCAAGAAGGTGGTGAAGCAG

Reverse CTTACTCCTTGGAGGCCATGT

Actin Forward GTCCCTGTATGCCTCTGGTC

Reverse CGCTCGGTCAGGATCTTCAT

Tubulin Forward AATGTCGGCCACCTTCATTG

Reverse CTCAGCCTCAGTGAACTCCA

Real Time PCR

mSRSF1 E2 Forward GAGTGGTTGTCTCTGGACTG

E3 Reverse TCTTCTTTCCGTACAAACTCCA

hSRSF1 E1E2 Forward GAGTTCGAGGACCCGCGAGACG

E2 Reverse GAGCTCCGCCACCTCCAC

Tubulin Forward AATGTCGGCCACCTTCATTG

Reverse CTCAGCCTCAGTGAACTCCA

mINSR Inc E10E11 Forward AGGAGCTGGAGGAGTCTTCA

Inc E10E11 Reverse CTACTGTCCTCGGCACCATT

mINSR Total INSR E1E2 Forward GCTGTGCCATTGCTGGTG

Total INSR E1E2 Reverse CAGCTCATGTAGCCTGGTCA

hINSR Inc E10E11 Forward CCTGAAGGAGCTGGAGGAGT

Inc E10E11 Reverse TAGGGTCCTCGGCACCAGT

hINSR Total INSR E1E2 Forward CTGTACCCCGGAGAGGTGT

Total INSR E1E2 Reverse GGGCCTCGTTTTGAACATC

Actin Forward GTCCCTGTATGCCTCTGGTC

Reverse CGCTCGGTCAGGATCTTCAT

Tubulin Forward AATGTCGGCCACCTTCATTG

Reverse CTCAGCCTCAGTGAACTCCA

hActin Forward GGCACCCAGCACAATGAAGA

Reverse AGGATGGAGCCGGCGATC

siRNAs

Luciferase. Anti-Luc siRNA 1 from Dharmacon. Catalog number (D-002050-01-20).

Target sequence GAUUAUGUCCGGUUAUGUA

SRSF1#1. From Sigma. Product Number NM_001078166.

SiRNA ID: SASI_Hs02_00313261.

Target Sequence: Sense CAUGUCUGAAGAUAGAUGA[dT][dT]

Antisense UCAUCUAUCUUCAGACAUG[dT][dT]

SRSF1#2. From Sigma. Product Number NM_001078166.

SiRNA ID: SASI_Hs02_00313262.

Target Sequence: Sense CAUCUACGUGGGUAACUUA[dT][dT]

Antisense UAAGUUACCCACGUAGAUG[dT][dT]
